# Technical Changes in Paraspinous Muscle Flap Surgery Have Increased Salvage Rates of Infected Spinal Wounds

**Published:** 2008-10-15

**Authors:** Alexander F. Mericli, John H. Moore, Steven E. Copit, James W. Fox, Gary A. Tuma

**Affiliations:** Division of Plastic and Reconstructive Surgery, Department of Surgery, Thomas Jefferson University Hospital, Philadelphia, PA

## Abstract

**Objectives:** The objective of this study is to introduce modifications in paraspinous muscle flap surgery and compare this new variation's ability to salvage infected hardware with the classic technique. Infected posterior spine wounds are a difficult problem for reconstructive surgeons. As per experience, hardware retention in infected wounds maintains spinal stability, decreases length of stay, and decreases the wound healing complication rate. **Methods:** An 11-year retrospective office and hospital chart review was conducted between July 1996 and August 2007. All patients who underwent paraspinous muscle flap reconstruction for postspine surgery wound infections during this time period were included. There were 51 patients in the study representing the largest reported series, to date, for this procedure. Twenty-two patients underwent treatment using the modified technique and 29 patients were treated using the classic technique. **Results:** There was no statistical difference between the 2 groups in demographics, medical history, or reason for initial spine surgery. The hardware salvage rate associated with the modified technique was greater than the rate associated with the classic technique (95.4% vs 75.8%; *P* = .03). There were fewer postreconstruction wound healing complications requiring hospital readmission in the modified technique group than the classic group (13.6% vs 44.8%; *P* = .04). Patients in the modified technique group demonstrated a shorter mean length of stay than the patients in the classic group (23.7 days vs 29.7; *P* = .25). **Conclusions:** The modified paraspinous muscle flap technique is an excellent option for spinal wound reconstruction, preservation of spinal hardware, and local infection control.

## INTRODUCTION

Infected posterior spine wounds are a difficult problem for reconstructive surgeons. With the increased use of alloplastic materials in spine surgery and the fact that this patient population has a diminished wound healing capacity because of certain comorbidities, many spine surgery patients are at a high risk for postsurgical wound complications that could result in exposure of the spinal cord, spinal hardware, or bony anatomy and thus necessitate surgical reconstruction of the wound.^1^ As per experience, hardware retention in infected wounds maintains spinal stability, decreases length of stay, and decreases the wound healing complication rate.^2^,^3^ Oftentimes, these infected spine wounds exist in the setting of concomitant chemoradiation therapy, malnutrition, ischemic tissues, and other challenges known to impede wound healing. Therefore, at institutions that perform a high volume of spine surgery, the reconstruction of infected midline back wounds from these procedures is becoming a more frequent dilemma encountered by the plastic surgeon.

Because the infection rate after spinal internal fixation can approach 20%, wound reconstruction with muscle is preferable to provide adequate tissue coverage, obliteration of dead space, and salvage of infected spinal hardware.^4^–^8^ Anatomically, however, providing optimal muscle flap coverage in the region of the spine can be challenging. Several techniques have been developed, including the latissimus and trapezius flaps and the superior gluteal artery flap. The paraspinous muscle flap is an excellent choice for wound reconstruction following spine surgery for a variety of reasons: it is anatomical, mirroring the long and narrow geometry of most spinal wounds; it is bipedicled and therefore highly vascular; there is a short operative time required; and there is no need for an additional donor site incision.^9^ The paraspinous flap is especially suited for soft tissue reconstruction of infected wounds with spinal fixation; the bilateral muscle flaps easily advance medially to completely cover the hardware, thereby, providing a highly vascular wound bed. In addition, for patients with fusions, no muscle function is lost with paraspinous flaps because the muscles are no longer required for spine flexion and extension.

There are currently 3 main variations of the paraspinous flap described in the literature. Wilhemi et al^9^ describes the classic technique in which the medial paraspinous muscle perforators are first ligated then the medial portion of the muscle is elevated and advanced over the midline with overlapping of the muscles; the muscle bellies are then approximated using simple interrupted sutures. Hultman et al^1^ reports a technique that differs from that of Wilhemi et al^9^ in that the lateral and posterior portion of the paraspinous muscle is elevated and mobilized. These lateral fibers are then sutured together in the midline as if closing the pages of an open book. Third, Saint-Cyr et al^6^ advocates elevation of the medial portion of the paraspinous muscle with medial muscle flap advancement and closure in a vest-over-pants fashion.

In this article, we present a fourth variation of the paraspinous muscle flap, which utilizes a general surgery principle: the Lembert suturing technique. This technique is most commonly used as the subserosal suture in hand-sewn bowel anastomoses; however, we have adapted it to be used to imbricate the bilateral paraspinous muscles over the spine. Although the paraspinous muscle flap is a well-known technique and has been discussed in the literature, no study exists that specifically compares the hardware salvage ability of one variation compared with another. This retrospective review of spinal wound reconstruction encompasses an 11-year period and involves 51 patients; this represents the largest reported study, to date, regarding hardware salvage using any variation of the paraspinous muscle flap. The *purpose* of the study is two-fold: (1) to introduce the modified paraspinous muscle flap technique and (2) to compare its efficacy with hardware salvage to the classic paraspinous muscle flap technique.

## METHODS

From July 1996 to July 2007, 51 patients (31 males and 20 females) underwent surgery to provide soft tissue coverage of the spine, following wound infection, using the paraspinous muscle flap technique. Prior to the development of a wound infection, all patients in the study had undergone spinal fusion with titanium instrumentation. Twenty-two patients had paraspinous muscle flap surgery performed using the modified technique and 29 patients had paraspinous muscle flap surgery performed using the classic technique. The classic technique was performed on patients who were earlier in the series, whereas the patients who underwent the modified technique were more recent. All surgeries were performed at a single university hospital by 1 of 3 attending plastic and reconstructive surgeons. Patients were referred to the department of plastic surgery by orthopaedics or neurosurgery, following the development of complications resulting in a nonhealing wound, often with exposure of spinal instrumentation, alloplastic materials, or bone grafts. Office and hospital charts were analyzed retrospectively to determine demographics, medical history, indications for initial spine surgery, indications for wound reconstruction, operative technique, postreconstruction course, length of stay, and duration of follow-up. Prereconstruction risk factors for poor wound healing included a history of radiation exposure of the wound area, the presence of hypertension, diabetes, steroid use, paralysis, smoking, collagen vascular disease, malnutrition, obesity, anemia, and a history of more than 2 previous spine operations. *Obesity* was defined as having a body mass index (BMI) > 30 kg/m^2^, *overweight* as a BMI between 30 kg/m^2^ and 25 kg/m^2^, and *normal weight* as a BMI < 25 kg/m^2^. *Malnutrition* was defined as having an albumin level < 3.5g/dL or prealbumin < 18g/dL. *Anemia* was defined as a hemoglobin level < 10 g/dL. Albumin, prealbumin, and hemaglobin levels were measured no greater than 1 week before reconstruction. *Length of stay* was defined as the duration of the admission, in days, during which wound reconstruction was performed. The study was approved by our institutional review board before initiation. Inclusion criteria were met if the patient underwent paraspinous muscle flap surgery for wound reconstruction following the development of a postspine surgery wound infection during the defined study period. Those cases were excluded in which reconstruction was performed prophylactically at the time of initial spine surgery, if the patient had no spinal hardware, or if reconstruction was performed for any reason besides infection (massive seroma, tumor resection, etc). Major outcome measurements included hardware salvage rate and postreconstruction wound complications, namely infection, dehiscence, seroma, and hematoma.

Descriptive statistics were calculated, including frequencies for categorical and ordinal variables and means, standard deviations, and ranges for continuous variables. Univariate analyses included independent *t* tests for the continuous outcome measures and the Cochran-Armitage chi-squared test for categorical outcome measures. Statistical significance was set at a *P* value of < .05. SAS statistical software (SAS Institute Inc, Cary, NC) was used for analysis.

## RESULTS

Over the course of the study, we performed the paraspinous muscle flap procedure for the salvage of infected spinal hardware in 51 patients. In 22 of these patients, the modified technique was performed and in 29 patients the paraspinous muscles were approximated in the classic fashion, using simple interrupted sutures. The mean age for patients undergoing the modified technique was 58.1 years, whereas the mean age for patients undergoing the classic techniques was 56.2 years. To ascertain whether either group was predisposed to developing a wound healing complication after reconstruction, we examined the incidence of several risk factors for poor healing. These included a history of radiation to the wound area, the presence of hypertension, diabetes, steroid use, paralysis, smoking, collagen vascular disease, malnutrition, obesity, anemia, and a history of more than 2 previous spine operations. There was a high rate of several factors associated with poor wound healing, including obesity, hypertension, and malnutrition (Table 1). There was no statistically significant difference between the 2 groups in demographics, medical history, or incidence of wound healing risk factors (Table 1). The size and location of the wound was also similar between the 2 groups: 5.86 vertebrae in the modified group compared with 6.51 vertebrae in the classic group (*P* = .63). In the modified group, 50% of the wounds were in the cervicothoracic area and 64% were in the lumbar area; in the classic group, 58% of the wounds were in the cervicothoracic area and 65% were in the lumbar area.

Patients in both groups underwent initial spine surgery for a variety reasons. These included degenerative disk disease as the most common reason followed by stenosis, trauma and other emergent causes, neoplastic disease, and infection (Table 2). Infections necessitating initial spine surgery included abscesses and osteomyelitis. There was no statistically significant difference between the 2 groups regarding the reason for initial spine surgery (Table 2).

All patients had clinically infected spinal wounds prior to debridement and reconstruction with paraspinous flaps. Patients in both groups were infected with similar types of organisms. In the modified technique group (*n* = 22), the greatest proportion were infected with methicillin-sensitive *Staphyococcus aureus* (10; 45%) followed by methicillin-resistance *Staphylococcus aureus* (8; 36%); other infecting organisms included species of *Pseudomonas* (1), *Enterobacter* (1), *Escherichia coli* (1), *Bacteroides* (1), *Enterococcus* (2), and *Serratia* (1). Two of the 22 patients (9%) in the modified technique group had a polymicrobial infection. In the classic group (*n* = 29), the majority of patients were infected with methicillin-sensitive *Staphyococcus aureus* (12; 41%) followed by methicillin-resistance *Staphylococcus aureus* (10; 34%); other infecting organisms included species of *Enterococcus* (4), *Enterobacter* (2), *Klebsiella* (2), *E coli* (2), *Serratia* (1), *Proteus* (1), and *Acinetobacter* (1). Seven of the 29 patients (24%) in the classic group had a polymicrobial infection. The average duration of antibiotic therapy in the modified technique group was 40.3 days, and in the classic group it was 43.3 days. All patients in both groups had no signs of wound infection at last follow-up.

The 2 treatment groups exhibited differences in hardware salvage and wound complication rates. Postreconstruction wound healing complications in both groups include reinfection and seroma formation. There were 6 postreconstruction wound healing complications in the modified technique group compared with 9 in the classic group (Table 3). Patients in the modified technique group had a statistically significant fewer number of postreconstruction wound complications resulting in hospital readmission (Table 3). At last follow-up, only 1 patient in the modified technique group required postreconstruction hardware removal, whereas 6 patients in the classic group have required hardware to be removed. Therefore, the modified technique demonstrates a significantly higher hardware salvage rate (95.4%) than the classic technique (75.8%; Table 3). The mean length of stay was shorter in the modified technique group (23.7 days) than the classic group (29.7 days); however, the difference was not significant (*P* = .25). The mean duration of follow-up in both groups was similar and not statistically significant (modified technique = 89.9 days; classic = 99.9 days; *P* = .85). All patients had stable wounds at the time of discharge with adequate coverage of hardware, alloplastic materials, and bone grafts. Regarding long-term functional outcome, all ambulatory patients were able to return to their baseline level of activity and no patients sustained any permanent decline in neurologic function.

## DISCUSSION

The modified technique for paraspinous muscle flap surgery is based on several basic principles of wound healing: the establishment of an adequate blood supply to the wound, aggressive and effective debridement, and wide suction drainage. The cornerstone of the modified technique is the Lembert suture variation. This method of suturing was first introduced by Antoine Lembert, a French, 19th century surgeon, in 1826. The technique was developed as a means to carefully approximate the serosal surfaces of small bowel in hand-sewn anastomoses.^10^ This method of suturing is widely used, to this day, in bowel anatomoses as well as in various gynecologic surgeries. We have adopted this technique for this particular procedure because the geometry of the suture imbricates the paraspinous muscle into the wound defect, effectively obliterating the dead space around the spinal hardware.

**Figure 1:**
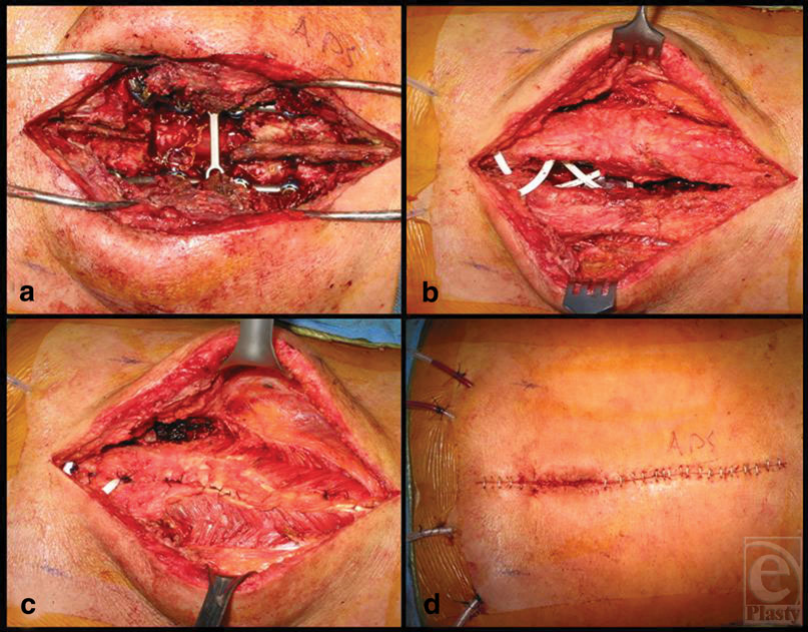
Step-wise progression of the modified paraspinous muscle flap technique. (a) The wound is aggressively debrided and irrigated. (b) The paraspinous muscles are elevated and 2 drains are placed in the submuscular space. (c) The paraspinous muscles are advanced medially and imbricated over the spine using the Lembert's technique. (d) Scarpas fascia, the deep dermis, and finally the skin are closed in a complex fashion.

**Figure 2:**
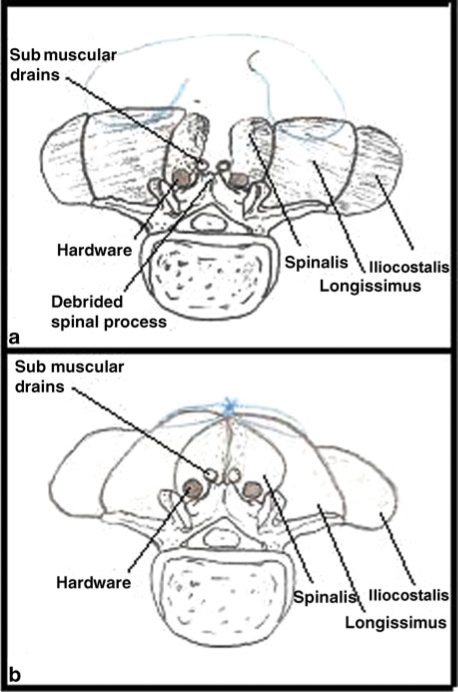
Sketches of the Lembert suturing technique used in the modified paraspinous muscle flap and the effect it has on the paraspinous musculature. (a) The suture enters the medial longissimus muscle, exits at the lateral aspect of the longissimus and then reenters the contralateral longissimus at the lateral aspect and exits at the medial aspect. (b) When the suture is pulled taught and tied, the medial spinalis portion of the musculature is forced into the deadspace surrounding the hardware creating a well-vascularized wound bed.

We first begin by aggressively debriding all skin, subcutaneous tissue, muscle, and bone in the wound bed (Fig 1a). All soft tissue is debrided using the Versajet Hydrosurgery System (Smith and Nephew, London, UK). Soft tissue is debrided until it appears healthy and well perfused. Special attention is paid to the medial edge of the paraspinous muscles. This tissue is frequently macerated and necrotic as a result of the initial instrumentation and subsequent infection. Each spinous process in the wound bed is debrided using a rongeur. Next, the wound is pulse lavaged with several liters of bacitracin-impregnated isotonic sodium chloride solution. After adequate irrigation, the subcutaneous tissue is elevated 5 to 7 cm over the paraspinous muscles, keeping the fascia intact. The fascia is then released lateral to each of the paraspinous muscles, allowing the bellies to advance medially. Two Blake drains are placed along the length of the wound in the submuscular space (Fig 1b). The paraspinous muscles are then imbricated over the drains using the interrupted Lembert suture technique with buried, no. 1 PDS sutures (Ethicon, Somerville, NJ, US). The suture enters the medial aspect of the longissimus muscle, travels laterally within the muscle, and exits at the lateral aspect of the longissimus; the suture then reenters the musculature at the lateral edge of the longissimus muscle on the contralateral side, travels medially within the muscle, and exits at the medial aspect of the longissimus muscle (Fig 2a). Special attention must be paid so that the suture is not allowed to exit the medial edge of the spinalis muscle, as would happen in the classic approach. When the suture is tied, the medial portion of the paraspinous muscle group (the spinalis muscle) is forced into the wound defect, obliterating the dead space around the hardware and creating a well-vascularized wound bed (Fig 2b). In the classic approach, the paraspinous muscles are simply approximated at the midline, tenting the musculature and leaving a large dead space between the muscle, vertebral body, and hardware. Finally, 2 more Blake drains are placed in the subcutaneous space. Scarpas fascia is closed with interrupted, buried, no. 1 PDS suture followed by 3.0 Monocryl (Ethicon, Somerville, NJ, US) in the deep dermis and finally staples on the skin (Fig 1d).

In comparing the modified technique group with the classic group, both patient groups demonstrated similar demographics and medical histories, therefore, it is unlikely that either group was predisposed to a higher rate of complications or hardware failure. The modified technique group had fewer postreconstruction wound healing complications requiring hospital readmission and a significantly higher hardware salvage rate (Table 3). Postreconstruction wound healing complications that did not require hospitalization included smaller seromas that could be safely drained in the office and minor infections. Hardware was removed after wound reconstruction only if the instrumentation loosened or if it did not become properly incorporated into the bone. These hardware complications are not necessarily mechanical in nature, but, rather, the wound bed must be well vascularized and devoid of inflammatory milieu for spinal instrumentation to become successfully incorporated into the vertebral bodies.^11^ Therefore, the higher hardware salvage rate associated with the modified technique, combined with the fewer number of reinfections, suggests that this new technique provides a superior wound reconstruction than the classic technique.

Compared with previously published studies on paraspinous muscle flap variations, the modified technique yields excellent results. Our data reveal a high hardware salvage rate (95.4%) and a low rate of wound healing complications requiring hospital readmission (13.6%; Table 3). The hardware salvage rate associated with the classic technique is 37.5% and wound healing complication rates range from 25% to 43%.^9^,^12^ The second reported variation, the vest-over-pants technique, was devised as a method to prevent cerebrospinal fistula formation. This study involved 9 patients and reported a wound healing complication rate of 22% and 1/1 hardware salvage rate.^6^ The Hultman technique is most similar to the modified technique in that both methods result in lateral approximation of the paraspinous muscles. Unlike the Hultman method, the modified technique features aggressive debridement via hydrodissection using the Versajet (Smith and Nephew, London, UK). Hultman et al^1^ details the outcomes of 25 patients, reporting a wound healing complication rate of 12% and a hardware salvage rate of 64%.^1^ Given the results from previously published studies, the modified technique appears to have a superior hardware salvage rate and a low rate of postreconstruction wound complications.

This study was inherently limited due to its retrospective design. Inevitably, there was recall bias on behalf of the researcher in collecting the data and on behalf of the surgeon, who was consulted when case clarification was necessary. To eliminate recall bias in the future, we plan to design a prospective study. In this study, patients that possess risk factors known to be associated with a significantly increased wound healing complication rate will be divided into 2 groups. One group will receive prophylactic paraspinous muscle flaps using the modified technique at the time of initial spine surgery and the other group will receive wound reconstruction on an as-needed basis. Later, we will compare the incidence of postreconstruction wound healing complications between the 2 groups. Providing prophylactic flap coverage in at-risk patients could potentially eliminate 2 surgeries: (1) paraspinous flap reconstruction after the development of a nonhealing wound and (2) operative debridement for postreconstruction wound healing complications. This may be an efficacious approach to spinal wound reconstruction in certain patient populations.

When compared with the classic group in this study and other published series of the paraspinous muscle flap procedure, the modified technique appears to improve the hardware salvage rate and decrease the number of wound complications. Furthermore, the modified technique is associated with a shorter mean length of stay than the classic technique. The paraspinous muscle flap remains an important tool in the reconstructive surgeon's armamentarium and this technical variation further improves on the flap's design, resulting in an improved wound healing ability.

## Figures and Tables

**Table 1:** Comparison of demographics and medical history between patient groups

	**Modified technique patients, *n* = 22, %**	**Classic technique patients, *n* = 29, %**	***P***
Mean age, y	58.1	56.2	.61
Obese	11 (50)	13 (45)	.71
Diabetes	9 (41)	8 (28)	.32
Hypertension	10 (45)	9 (31)	.29
Steroids	4 (18)	5 (17)	.93
XRT	2 (9)	2 (7)	.77
Malnutrition	17 (85)	23 (77)	.48
Anemia	12 (54)	14 (50)	.75
Paralysis	3 (14)	4 (14)	.98
Current smoker	2 (9)	1 (3)	.39
Former smoker	7 (32)	6 (21)	.36
Current or former smoker	9 (41)	7 (24)	.2
Collagen vascular disease	2 (9)	2 (7)	.77
History of more than 2 spine surgeries	2 (9)	5 (17)	.4
Emergent spine surgery	6 (27)	4 (14)	.23

**Table 2:** Initial spine surgery

	**Modified technique patients, *n* = 22, %**	**Classic technique patients, *n* = 29, %**	***P***
Degenerative disk disease	8 (36.3)	12 (41.1)	.71
Stenosis	6 (27.2)	8 (27.6)	.98
Neoplasm	4 (18.2)	3 (10.3)	.42
Emergent	6 (27)	4 (14)	.23
Infection	2 (9.1)	0 (0)	.09

**Table 3:** Postreconstruction salvage rate and wound healing complication rate

	**Modified technique patients, *n* = 22, %**	**Classic technique patients, *n* = 29, %**	***P***
Hardware salvage rate	21 (95.4)	22 (75.8)	.03
Infection	2 (9.1)	5 (17.2)	.40
Seroma	4 (18.2)	2 (6.9)	.21
Hematoma	0 (0)	0 (0)	0
Dehiscence	0 (0)	0 (0)	0
Complications requiring hospital readmission	2 (13.6)	9 (44.8)	.04
Total complications	6 (36.3)	9 (48.3)	.23
